# Antimicrobial activity of polyhexamethylene guanidine phosphate in comparison to chlorhexidine using the quantitative suspension method

**DOI:** 10.1186/s12941-015-0097-x

**Published:** 2015-07-17

**Authors:** A Vitt, A Sofrata, V Slizen, R V Sugars, A Gustafsson, E I Gudkova, L A Kazeko, P Ramberg, K Buhlin

**Affiliations:** Division of Periodontology, Department of Dental Medicine, Karolinska Institutet, Alfred Nobels Allé 8, Box 4064, 141 04 Huddinge, Sweden; Division of Oral Facial Diagnostics and Surgery, Department of Dental Medicine, Karolinska Institutet, Huddinge, Sweden; First Department of Therapeutic Dentistry, Belarusian State Medical University, Minsk, Belarus; Department of Medical Microbiology and Immunology, Belarusian State Medical University, Minsk, Belarus; Division of Periodontology, Institute of Odontology, Sahlgrenska Academy, University of Gothenburg, Gothenburg, Sweden

**Keywords:** Periodontal disease, Chlorhexidine, Polyhexamethylene guanidine phosphate

## Abstract

**Background:**

Polyhexamethylene guanidine phosphate (PHMG-P) belongs to the polymeric guanidine family of biocides and contains a phosphate group, which may confer better solubility, a detoxifying effect and may change the kinetics and dynamics of PHMG-P interactions with microorganisms. Limited data regarding PHMG-P activity against periodontopathogenic and cariogenic microorganisms necessitates studies in this area. Aim is to evaluate polyhexamethylene guanidine phosphate antimicrobial activity in comparison to chlorhexidine.

**Methods:**

Quantitative suspension method was used enrolling *Staphylococcus aureus*, *Pseudomonas aeruginosa*, *Escherichia coli* and *Candida albicans*, *Aggregatibacter actinomycetemcomitans*, *Porphyromonas gingivalis*, *Streptococcus mutans* and *Lactobacillus acidophilus*.

**Results:**

Both tested antiseptics at their clinically-used concentrations, of 0.2% (w/v) and 1% (w/v), correspondingly provided swift bactericidal effects against *S. aureus*, *P. aeruginosa*, *E. coli andC. albicans*, *A. actinomycetemcomitans* and *P. gingivalis* with reduction factors higher than 6.0. Diluted polyhexamethylene guanidine phosphate and chlorhexidine to 0.05% continued to display anti-bacterial activity and decreased titers of standard quality control, periopathogens to below 1.0 × 10^3^ colony forming units/ml, albeit requiring prolonged exposure time. To achieve a bactericidal effect against *S. mutans*, both antiseptics at all concentrations required a longer exposure time. We found that a clinically-used 1% of polyhexamethylene guanidine phosphate concentration did not have activity against *L. acidophilus*.

**Conclusion:**

High RF of polyhexamethylene guanidine phosphate and retention of bactericidal effects, even at 0.05%, support the use of polyhexamethylene guanidine phosphate as a biocide with sufficient anti-microbial activity against periopathogens. Polyhexamethylene guanidine phosphate displayed bactericidal activity against periopathogens and *S. mutans* and could potentially be applied in the management of oral diseases.

**Electronic supplementary material:**

The online version of this article (doi:10.1186/s12941-015-0097-x) contains supplementary material, which is available to authorized users.

## Background

Dental plaque, is a biofilm that is recognized as a cause and significant risk factor for periodontal diseases [1]. The imbalance in non-pathogenic biofilm auto-regulation, behavioral aspects and immunological reactivity of the host results in maximizing the pathological potential of dental plaque with the accumulation of periopathogens, and an increased risk of periodontal disease. The pathogenic nature of the dental biofilm can be diminished in the oral cavity by reducing the bioburden and effectively maintaining a normal oral flora via oral hygiene routines (daily toothbrushing and flossing) and treatment procedures (scaling and root planning) [2, 3]. To enhance the efficacy of periodontal treatment, locally delivered anti-microbials have become an essential component of anti-infective management of periodontal diseases and has stimulated the development of state-of-the-art antiseptics for local application to modify biofilm composition, and re-establish dental biofilm autoregulation [4]. Mature biofilm shows a higher tolerance for anti-microbial agents [5]. Thus, it has been suggested that minimal inhibitory concentrations should be determined for bacteria as part of a biofilm and not in the planktonic state [6]. However, currently there is lack of standardized methods to perform this type of assessment [7]. The properties of the biofilm can differ depending on the environment on which it forms. For example, *Acinetobacter baumannii* has different resistance profiles in laboratory media versus on ex vivo human ascites [8].

A considerable amount of research has concentrated on evaluating cellular toxicity and bacterial activity of topical anti-microbials to derive arrays of biocompatible antiseptics that can be exploited in dentistry for anti-infective management of periodontal diseases. Selection of antiseptics is based on their specificity, efficacy, substantivity, safety, stability and plaque control. Of the miscellaneous antiseptics, chlorhexidine (CHX) has acquired a widespread and successful application in dentistry and has been considered the golden standard for many years [9]. Some adverse effects of CHX, include tooth and tongue staining, changes in taste, desquamation of the mucosa membrane in the oral cavity, a dose dependent reduction in collagen or non-collagenous protein production by gingival fibroblasts, and a reduction in their proliferation [10]. These have given an impetus for the development new antiseptics without adverse effects for administration in dentistry.

Polyhexamethyleneguanidine (PHMG) derivates are members of the polymeric guanidine family that have been widely used for many years as antiseptics in medicine and the food industry [11]. Detoxified conjugates of polyhexamethylene biguanide hydrochloride (PHMB-H) and polyhexamethylene guanidine phosphate (PHMG-P) with incorporated different anions are highly soluble in water [12]. PHMB-H has been extensively tested in vivo and in vitro [11, 13–15]. Clinical studies have shown that PHMB-H mouthwash consistently inhibits plaque regrowth and reduces oral bacterial counts, indicating that PHMB-H could be an alternative active substance of dentifrices. Data regarding specificity, efficacy, substantivity, safety, stability and plaque control of PHMG-P are unavailable and its efficacy in the treatment of periodontal diseases and activity against periodontopathogenic and cariogenic bacteria requires evaluation. Following antiseptic administration in the oral cavity, over time antiseptics are gradually diluted in the saliva and their activity decreases from the start of treatment [16]. Thereafter, antiseptics even at low concentrations are able to continue exerting effects until inactivation. Therefore, anti-microbial activity should be tested not only on the clinically-used working concentration but on several dilutions with different exposure times.

In this study the bactericidal effects of PHMG-P have been studied on planktonic forms of standard bacterial species along with cariogenic and periodontopathogenic bacteria to evaluate its feasibility in the management of carries and periodontal diseases. The aim of this study was to evaluate the prospective of application of PHMG-P based antiseptics in dentistry by comparing PHMG-P anti-microbial activity against periopathogenic, cariogenic and standard quality control microorganisms to CHX using the quantitative suspension method.

## Methods

### Bacterial strains and cultivation

Anti-bacterial activity of antiseptics was estimated on the following standard strains of *Staphylococcus aureus* (ATCC 6538), *Escherichia coli* (ATCC 11229), *Pseudomonas aeruginosa* (ATCC 15412), and *Candida albicans* (ATCC 1023), and on the oral Gram-negative periodontal pathogens *Porphyromonas gingivalis* (ATCC 33277), *Aggregatibacter actinomycetemcomitans* (HK 1519), Gram-positive cariogenic strains *Streptococcus mutans* (CCUG 27624; Ing-Brit), and *Lactobacillus acidophilus* (NCTC 1723). The strains of *S. aureus*, *E. coli*, *P. aeruginosa* and *C. albicans* were inoculated on trypticase soy agar (TSA) (Becton–Dickinson, NJ, USA) and incubated for 18–24 h at 37°C. The number of these microorganisms suspended in sterile phosphate buffered saline (PBS) was adjusted by detection of optical (OD) density and determined precisely by the drop count method. *P. gingivalis* was cultured on Colombia base agar (Acumedia, Baltimore, MD, USA) supplemented with hemin (0.05 mg/ml) (Sigma-Aldrich, Sweden AB), vitamin K (0.01 mg/ml) (BBL™, Becton–Dickinson), and citrated horse blood (5%) (Sigma-Aldrich), in anaerobic atmosphere (GasPak, Becton–Dickinson) for 7 days. The test-suspension of *P. gingivalis* with OD 0.73–0.75 was prepared in peptone yeast glucose broth (Becton–Dickinson). Two days culture of *A. actinomycetemcomitans* grown on Colombia base agar supplemented with 0.01% tryptophan and citrated horse blood (5%) was suspended in haemophilus teat medium (HTM) broth (Bacto™, Becton–Dickinson). OD of the suspension was adjusted to 0.76–0.78. *S. mutans* were cultured on brain heart infusion (BHI) (Oxoid, Malmö, Sweden) agar for 2 days and a suspension with an OD of 0.14 was prepared in BHI broth. *L. acidophilus* was cultured on *Lactobacilli* MRS (de Man, Rogosa and Sharpe) medium (Difco™, Becton–Dickinson) for 2 days and a test-suspension with an OD of 0.14–0.15 was prepared in *Lactobacilli* MRS broth. The reference strains of *S. mutans*, *A. actinomycetemcomitans*, *L. acidophilus* were cultured in capnophilic atmosphere containing 5% CO_2_. The turbidity of all suspensions was standardized for each bacterial strain using a spectrophotometer at 580 nm (Biochrom WPA CO7500 Colorwave Colorimeter), to provide the concentration of test-microorganisms approximately equal to 1 × 10^8^ colony-forming unit/ml (CFU/ml). All bacteria were filtered through 5 μm (Pall Corporation, USA) to provide better separation.

### Antiseptics

A working concentration of PHMG-P 1% (w/v), typically used in the clinic setting, and dilutions 0.5, 0.2, 0.05% (w/v) were prepared *ex tempore* in dH_2_O from concentrated 70% PHMG-P gel, with an average molecular weight of 4,000–9,000 (Institute of Eco-Technological Problems, Moscow, Russia). Anti-microbial activity of PHMG-P was tested in comparison to 0.05, 0.2, 0.5, 1% (w/v) CHX prepared from 1% CHX (Apotek Produktion and Laboratorier AB, Stockholm, Sweden). Each concentration of antiseptic (PHMG-P and CHX) was exposed for 30 s, 1, 3, and 5 min, respectively. Hydrogen peroxide (H_2_O_2_) 3% (w/v) was included, as a control for a product with known efficacy.

### Neutralizing solution

Neutralizing solution, encompassing 1% (w/v) peptone, 3% (w/v) Tween 80 (BDH, Poole, UK), 0.3% (w/v) lecithin (Fisher Scientific, Loughborough, UK), 0.1% (w/v) histidine (BDH) and 0.1% (w/v) cysteine (Sigma-Aldrich) was prepared in dH_2_O and sterilized.

### Bactericidal activity evaluation

Bactericidal activity of anti-microbials was tested by using the quantitative suspension method according to European standards EN 1040:2005 CSN EN 1040 at room temperature. Briefly, 50 μl of the test-suspensions of microorganisms with an OD of 1.0 × 10^8^ CFU/ml were exposed to 450 μl of each antiseptic (PHMG-P, CHX or H_2_O_2_) at concentrations of 0.05, 0.2, 0.5, and 1% for 30 s, 1, 3 and 5 min, followed by transfer of the mixture to 450 μl neutralizer. After neutralization for 10 min a serial dilution (10^−3^, 10^−4^, and 10^−5^) was performed and 100 μl aliquots were plated on appropriate media and incubated at 37°C for 2–7 days for respective bacteria. Colonies were counted and the viability of the test mixture calculated. Anti-bacterial properties of neutralizing solution to test-microorganisms were controlled. Activity of antiseptics was evaluated by reduction factor (RF) that was calculated as the difference between logarithms of CFU/ml before and after exposure to tested concentrations of antiseptics. Sensitivity threshold of the method was considered as 1.0 × 10^3^ CFU/ml of bacteria. If the antiseptic led to total loss of bacteria, defined as no bacterial growth, it was assigned as ≤1.0 × 10^3^ CFU/ml. Detection of bactericidal effect of the antiseptic agents against test species was performed on two separate occasions in duplicate, for each product and each exposure time.

Activity of PGMG-P versus CHX was evaluated on the basis of observed bactericidal effect resulting in a total loss of bacteria and a titer beneath 1.0 × 10^3^ CFU/ml. Such pairwise comparison did not allow for the estimation of difference in CFU number.

## Results

### Activity of CHX and PHMG-P against standard quality-control microorganisms

**Figure 1:**
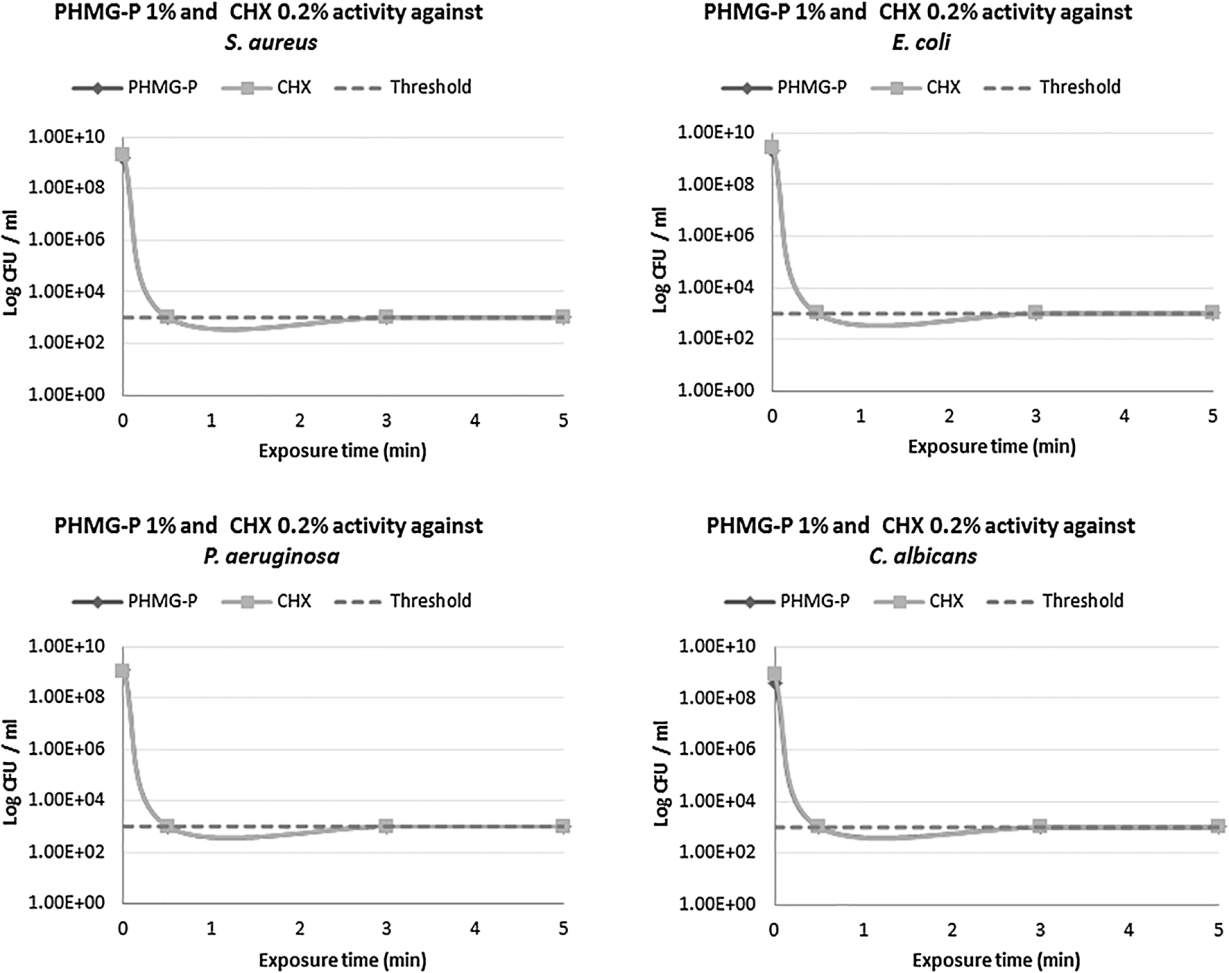
Anti-microbial activity of clinical working concentrations of PHMG-P (1%) and CHX (0.2%) against standard quality control microorganisms (*S. aureus*, *E. coli*, *P. aeruginosa* and *C. albicans*).

**Figure 2:**
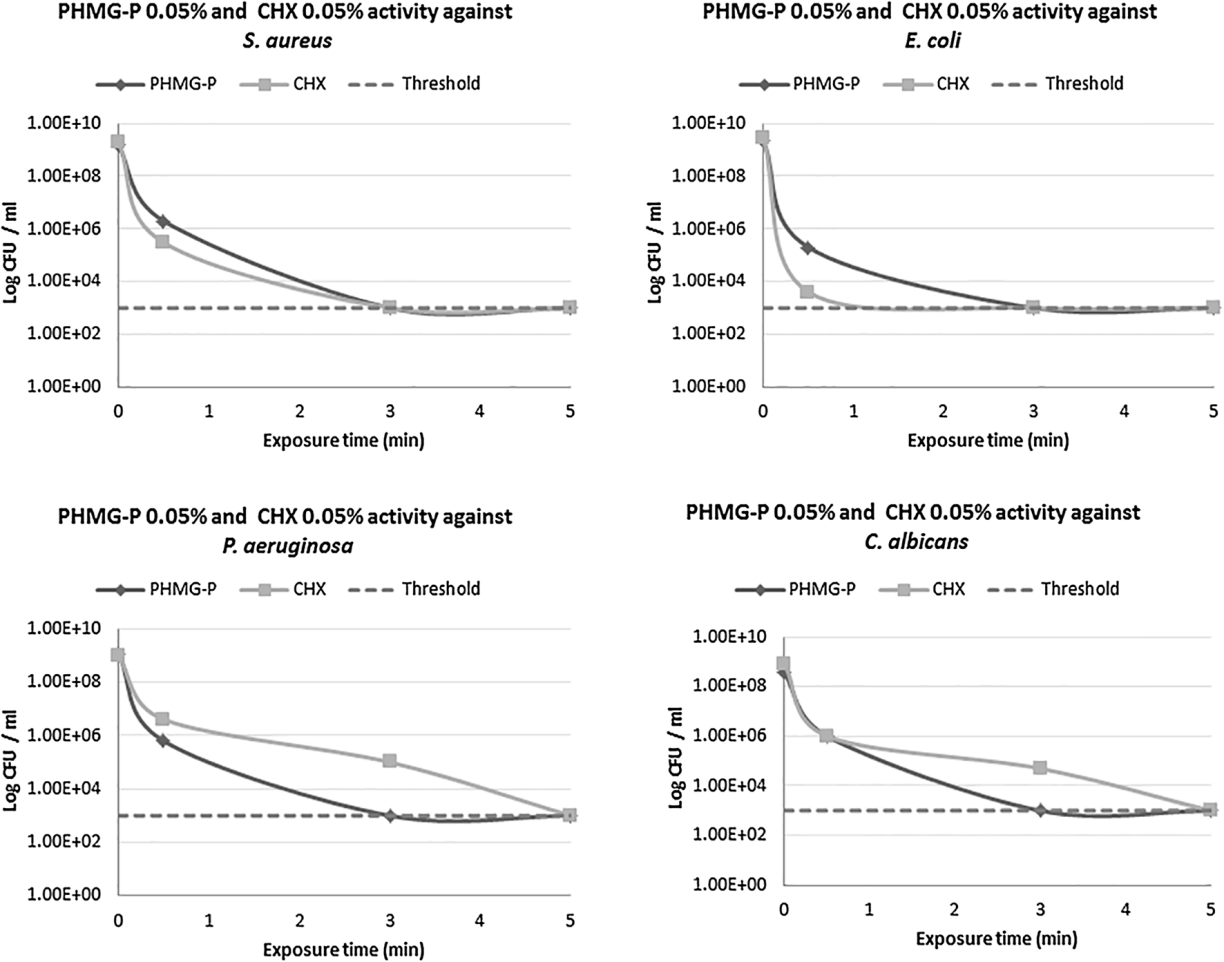
Anti-microbial activity of highly diluted PHMG-P (0.05%) and CHX (0.05%) against standard quality control microorganisms (*S. aureus*, *E. coli*, *P. aeruginosa* and *C. albicans*).

Anti-microbial properties of PHMG-P were tested in comparison with CHX against quality-control strains of *S. aureus*, *P. aeruginosa*, *E. coli*, *C. albicans*. Results are summarized in Figures 1, 2, and Additional file 1: Table S1.

Anti-microbial action of clinically-relevant working concentrations of PHMG-P (1%) and CHX (0.2%) against standard quality control microorganisms (*S. aureus*, *E. coli*, *P. aeruginosa* and *C. albicans*) exhibited a very similar pattern of activity (Figure 1). 1% PHMG-P and 0.2% CHX expressed rapid bactericidal effects on all species within 30 s of action and decreased the bacterial titer below 1.0 × 10^3^ CFU/m (RF over 5).

Both 0.05% diluted PHMG-P and CHX (20-fold) were unable to eliminate *S. aureus* over 30 s exposure and led to a reduction of bacterial titer from 1.47 × 10^9^ to 1.8 × 10^4^ CFU/ml and from 2.0 × 10^9^ to 3.0 × 10^5^ CFU/ml, respectively (Figure 2). Prolonged biocide exposure for 3 min caused a strong bactericidal effect, accompanied by a falling microbial titer below the sensitivity threshold.

Application of 0.05% PHMG-P and 0.05% CHX for 30 s decreased the *E. coli* population from 2.17 × 10^9^ to 2.0 × 10^5^ CFU/ml (RF 4.04), and from 2.7 × 10^9^ to 4.0 × 10^3^ CFU/ml, respectively, whilst increased treatment time to 3 min resulted in total eradication of bacteria (RF 6.48 and 6.41 correspondingly) (Figure 2).

Only extended exposure of *P. aeruginosa* for 3 min at 0.05% PHMG-P resulted in a full bacteria elimination (RF 6.07), however bactericidal activity against *P. aeruginosa* with 0.05% CHX required 5 min exposure (RF 6.0) (Figure 2).

Application of 0.05% PHMG-P and 0.05% CHX for 30 s exposure to *C. albicans* did not produce any significant anti-candidal effect and reduced fungal population from 3.77 × 10^8^ to 1.0 × 10^6^ CFU/ml and from 8.0 × 10^8^ to 1.0 × 10^6^ CFU/ml, respectively (Figure 2). 0.05% PHMG-P eliminated *C. albicans* within 3 min, whilst 0.05% CHX decreased the amount of fungi below 1.0 × 10^3^ CFU/ml only after longer treatment period (5 min).

### Activity of CHX and PHMG-P against perio- and cariogenic microorganisms

**Figure 3:**
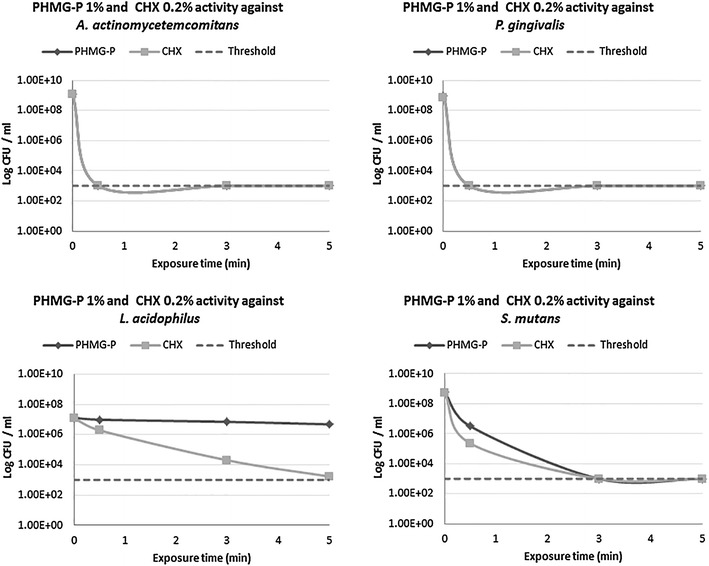
Anti-microbial activity of clinical working concentrations of PHMG-P (1%) and CHX (0.2%) against perio- and cariogenic bacteria (*P. gingivalis*, *A. actinomycetemcomitans*, *S. mutans* and *L. acidophilus*).

**Figure 4:**
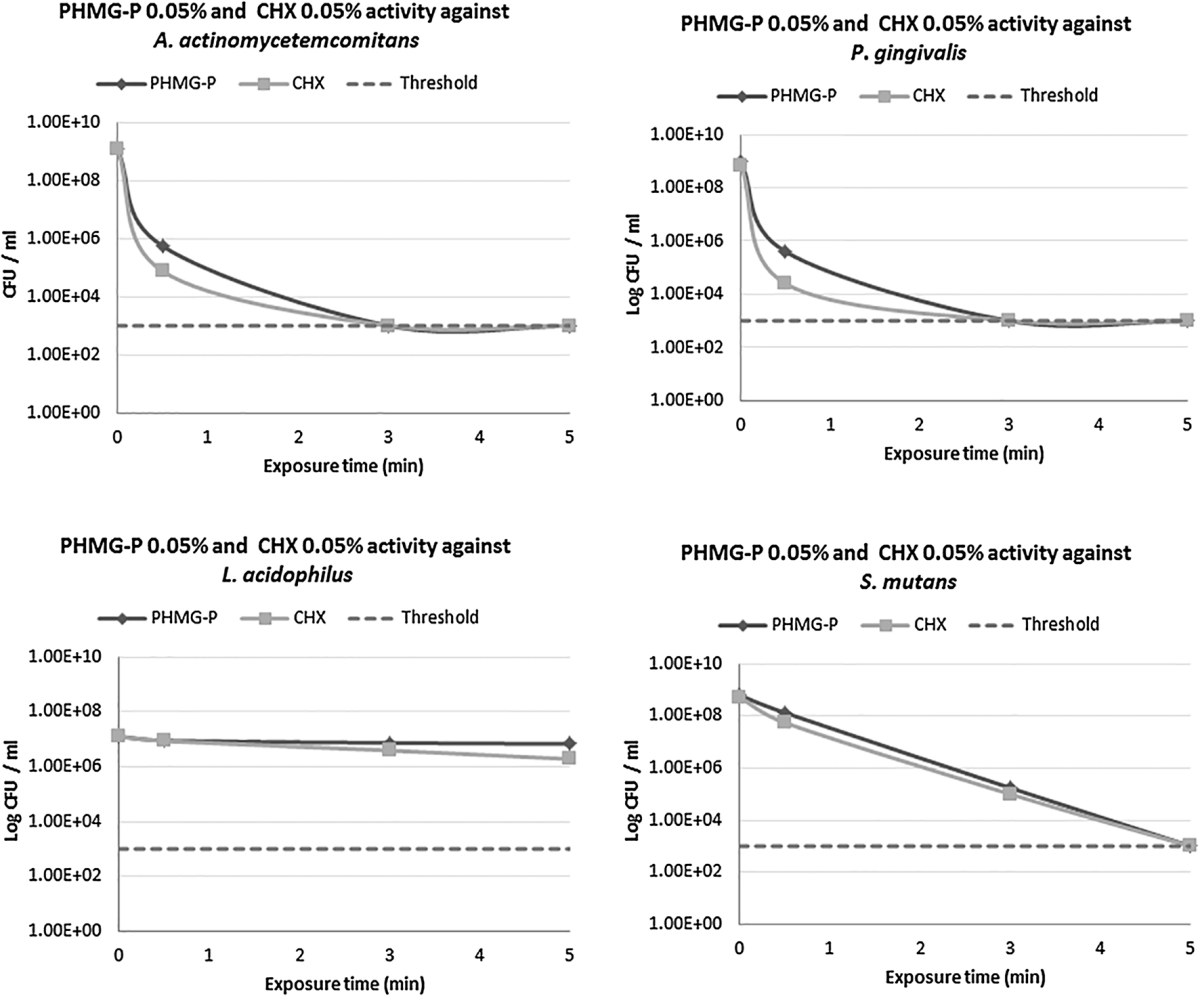
Anti-microbial activity of highly diluted PHMG-P (0.05%) and CHX (0.05%) against perio- and cariogenic bacteria (*P. gingivalis*, *A. actinomycetemcomitans*, *S. mutans* and *L. acidophilus*).

The results of antimicrobial activity of antiseptics against periopathogenic and cariogenic microorganisms are shown in Figures 3, 4, and Additional file 2: Table S2.

1.0% PHMG-P and 0.2% CHX, clinically-relevant working concentrations, effectively inhibited periopathogens (*A. actinomycetemcomitans* and *P. gingivalis*) (Figure 3), displaying swift activity within 30 s of exposure with a decrease in microbial titers less than 1 × 10^3^ CFU/ml, for each periopathogen resulting in RF’s over 6.06 and 5.86, respectively.

Evaluation of 1% PHMG-P and 0.2% CHX bactericidal activity against *S. mutans* indicated that tested concentrations of antiseptics after 30 s exposure did not result in any significant reduction in the concentration of *S. mutans*, with RF’s equaling 2.3 and 3.88 (Figure 3). Following an extended treatment time (3–5 min), the working concentration of PHMG-P (1%) and CHX (0.2%) completely inhibited *S. mutans*. Thus, the titer of these cariogenic microorganisms also fell from below 1 × 10^3^ CFU/ml (RF’s 5.8 and 5.73).

Highly diluted PHMG-P and CHX (0.05%) acting for 30 s exhibited a milder influence against *A. actinomycetemcomitans* and *P. gingivalis*, characterized by RF’s lower than 4 (Figure 4). Extending antiseptic treatment for 3 min resulted in total bacteria elimination (RF ≥ 5.86) for both reagents.

PHMG-P and CHX at concentrations of 0.05% over 30 s caused only a minor fall in microbial concentration of *L. acidophilus*, with RF values 0.24 and 0.51, respectively (Figure 4). Increased antiseptic concentrations of 1% PHMG-P and 0.2% CHX after 5 min exposure failed to produce a bactericidal effect (Figure 3). The RF value of PHMG-P with respect to *L. acidophilus* varied in the range of 0.12–0.43 suggesting extremely low anti-bacterial activity. However, CHX expressed higher anti-microbial activity against *L. acidophilus* and at concentrations of 0.5% for 5 min resulted in total bacteria eradication (RF 4.1). Unlike periopathogens, stricter PHMG-P and CHX application modes were required to control *L. acidophilus* cell titers below 1.0 × 10^3^ CFU/ml.

In the present study, PHMG-P and CHX at 0.05% for 30 s displayed only limited anti-microbial impact on *S. mutans*, illustrated by the respective RF values of 0.28 and 0.99, respectively (Figure 3). 0.05% PHMG-P and 0.05% CHX over 3 min also demonstrated insufficient anti-microbial activity (RF 3.55 and 3.73 correspondently), when extended to 5 min treatment, a reduced amount of *S. mutans* below the methods sensitivity threshold (≤1 × 10^3^ CFU/ml) was obtained.

3% H_2_O_2_ eliminated *S. aureus*, *E*. *coli* and *P. aeruginosa* within 30 s of exposure time (RF’s ≤ 6.29). Prolonged biocide exposure for 5 min was required for H_2_O_2_ to eradicate *C. albicans* (RF 5.42). In addition, control of neutralization confirmed absence of anti-bacterial effect of neutralizer on bacteria since no fall CFU/ml was registered after neutralization (data not shown).

## Discussion

Comparative anti-microbial activity of antiseptic agents using suspension-based methods, broth or agar dilution have been evaluated in many studies and the data regarding anti-bacterial efficacy of antiseptics are contradictive [15, 17, 18]. In clinical studies, concentrations of 0.04 and 0.12% PHMB-H mouthwash were shown to inhibit plaque re-growth and reduce oral bacterial counts [11, 13]. However, both PHMB-H concentrations were less effective than 0.12% CHX positive control rinse. Further studies support the ability of PHMB-H, at a higher concentration (0.2%), to diminish oral bacterial counts and prevent plaque re-growth, but this was significantly lower compared to CHX [14]. In vitro studies have concluded that CHX-based mouthwashes presented better anti-microbial activities against *S. aureus* than the PHMB-based mouthwash [19]. In contrast, it has been reported that at 0.002% CHX did not eliminate *P. gingivalis*, *A. actinomycetemcomitans*, *Fusobacterium nucleatum*, *Tannerella forsythensis*, *Prevotella intermedia and Streptococcus anginosus* after 1 min exposure time, unlike the anti-microbial effects 0.23% povidone-iodine (PVP-I) that eliminated bacteria after 15 s [17]. Müller claimed that polyhexamethylene bisguanide (PHMB) 7,000 mg/L had a stronger anti-bacterial efficacy than CHX 100 mg/L, which in turn exceed PVP-I (7,000 mg/L), against *S. aureus* and *E. coli* [15].

In light of these apparent conflicts, the present study, showed both PHMG-P and CHX to have similar significant anti-microbial activities against standard quality control microorganisms and periopathogens. A lack of rapid bactericidal of PHMG-P effect against *S. mutans* over 30 s was apparent and required an increased duration of exposure and repeated application to achieve the desired effect in the course of prophylactic or therapeutic procedures. As opposed to 1% CHX, 1% PHMG-P did not exhibit bactericidal effects against *L. acidophilus* within 5 min of contact, which does not mean that the substance was inactive. PHMG-P gradually decreased *L. acidophilus* titers in a time and concentration dependent manner, however method sensitivity threshold was not reached.

Minimal concentrations of CHX decreased the amount of majority of bacteria (*S. aureus*, *E. coli*, *P. aeruginosa*, *A*. *actinomycetemcomitans* and *P. gingivalis*) to below detective limits of 0.2%, and increasing the concentration did not improve efficacy. For PHMG-P, such minimal effective concentration was 0.2% for *E. coli* and *P. aeruginosa* and 0.5% for *S. aureus*, *A.actinomycetemcomitans* and *P. gingivalis*. CHX expressed activity at lower concentrations for 30 s against *S. aureus*, *A*. *actinomycetemcomitans* and *P. gingivalis*, however the working concentration equals the minimal effective concentration (30 s) left a narrow therapeutic window for the antiseptics express their maximum effect before dilution in saliva.

Studies in plant physiology, confirm that fructan, for example levan has a direct protective effect and capacity to stabilize membranes during drying by inserting part of the polysaccharide into the lipid headgroup region [20]. It is well-established that *Lactobacilli* produce a broad range of polymers, including levan- and inulin-type fructans and α-glucans (dextran, mutan and reuteran). Homo-polysaccharide and oligosaccharide production are most frequently found in *L. acidophilus*, *L. johnsonii*, *L. mucosae*, and *L. reuteri* [21]. Some species of lactobacilli display an additional peptidoglycan outer paracrystalline layer of proteins (S-layer), which is a two-dimensional array of protein or glycoprotein subunits with stable tertiary structures ranging from 40 to 60 kDa, highly basic, assembled in lattices with different symmetries that represent 10–15% of total cell wall proteins [21, 22]. Basic character of S-layer proteins might influence the electrostatic interaction with cationic PHMG-P. This is accompanied by lack of bactericidal effect of PHMG-P on *L. acidophilus*. Perhaps, the decreased concentration of *Lactobacilli* in adults cannot be considered as an important factor in the prevention of dental caries, because 69% of tested *Lactobacilli* were reported to inhibit the growth of *S. mutans*, 88% of *A. actinomycetemcomitans*, 82% of *P. gingivalis* and 65% of *Prevotella intermedia* [23]. Despite the fact that PHMG-P selectively acts against *S. mutans* and has no effect on *L. acidophilus* this biocide may be regarded as a preventive remedy for dental caries.

According to our data, PHMG-P at concentration of 0.05% required 3 min to achieve bactericidal effects against the majority of bacteria. Thus, delayed bactericidal activity kinetics may be correlated with the three-dimensional structure of PHMG-P. Literature reports, closely related PHMG-H, to possess seven types of molecular structures, including three linear types and four cyclic or branched ones, with an average coefficient of branching of 0.16–1.08 per molecule [24]. It has been established for PHMG-H that anti-microbial activity increased on a mass basis with elongated polymer chain length, high molecular weight materials with n >10 were highly effective.

Evaluation of bactericidal effect on different water-soluble polymeric derivates of guanidine showed their good anti-microbial activity towards bacteria at low doses (13 mg/ml) [25]. They slightly damaged the outer membrane layer in cell envelope of *E. coli* and increased the permeability of the cytoplasmic membrane, whilst no significant damage was observed in the morphological structure of the cells. High doses (23 mg/ml) of PHMG-H caused collapse of the outer membrane structure, generating local pores across the membrane and inflicting severe lesions of the internal cell structure, leakage of intracellular components and cell inactivation [25]. PHMG-H containing disinfectant “Akwaton” displayed a sporicidal effect against suspended and fixed spores of *Bacillus subtilis* exposed for 1.5 min contact time ≥0.44% (w/v) and 0.52% (w/v), respectively [26]. Akacid plus (consisting of a mixture (3:1) of PHMG-chloride and poly-[2-(2-ethoxy)-ethoxyethyl)-guanidinium-chloride]) removed *S. aureus*, *Enterococcus hirae*, *E. coli*, *P. aeruginosa*, *C. albicans* and *Aspergillus niger* at 0.1% within 5 min [27]. PHMG-H based disinfectant displayed its germicidal function at very low concentrations, 0.005% (w/v) for *E. coli* and 0.04% (w/v) for MRSA, eliminating bacteria by 1.5 min [28].

## Conclusion

The substance PHMG-P displayed anti-bacterial activity against standard quality control strains, periopathogens and *S. mutans*, even after a 20-fold dilution. The use of the antiseptic could have potential applications in the management of oral diseases, such as caries and periodontal diseases.

## Additional files

Additional file 1:
**Table S1.** Anti-microbial activity of antiseptics against standard quality-control microorganisms in the quantitative suspension method. Additional file 2:
**Table S2.** Anti-microbial activity of antiseptics against perio- and cariopathogens in the quantitative suspension method.
