# Supplementary material for: Antimicrobial activity of polyhexamethylene guanidine phosphate in comparison to chlorhexidine using the quantitative suspension method
Source: Ann Clin Microbiol Antimicrob. 2015 Jul 17;14:36. doi: 10.1186/s12941-015-0097-x (PMC4504446; doi:10.1186/s12941-015-0097-x)
Supplement: Additional file 1: — Table S1. Anti-microbial activity of antiseptics against standard quality-control microorganisms in the quantitative suspension method. [file 12941_2015_97_MOESM1_ESM.pdf]

Table S1. Anti-microbial activity of antiseptics against standard quality-control microorganisms in the quantitative suspension method

**Test-culture**	**Exposure time, (min)**	**Polyhexamethylene guanidine phosphate (PHMG-P) concentration, %**									**Chlorhexidine (CHX) concentration, %**									**Hydrogen peroxide (H_2O_2) concentration, %**		
		Control	1.0		0.5		0.2		0.05		Control	1.0		0.5		0.2		0.05		Control	3.0	
		CFU/ml	CFU/ml	RF	CFU/ml	RF	CFU/ml	RF	CFU/ml	RF	CFU/ml	CFU/ml	RF	CFU/ml	RF	CFU/ml	RF	CFU/ml	RF	CFU/ml	CFU/ml	RF
S. aureus	0.5	1.47×10^{9}	1.0×10^{3}	6.17	1.0×10^{3}	6.17	$1.8\cdot10^{4}$	4.91	2.0×10^{6}	2.87	2.0×10^{9}	1.0×10^{3}	6.3	1.0×10^{3}	6.3	1.0×10^{3}	6.3	$3.0\cdot10^{5}$	3.83	2.25×10^{9}	1.0×10^{3}	6.35
ATCC 6538	3.0	$1.62\cdot10^{9}$	1.0×10^{3}	6.21	1.0×10^{3}	6.21	1.0×10^{3}	6.21	1.0×10^{3}	6.21	2.0×10^{9}	1.0×10^{3}	6.3	1.0×10^{3}	6.3	1.0×10^{3}	6.3	1.0×10^{3}	6.3	2.15×10^{9}	1.0×10^{3}	6.33
	5.0	$1.6\cdot10^{9}$	1.0×10^{3}	6.2	1.0×10^{3}	6.2	1.0×10^{3}	6.2	1.0×10^{3}	6.21	2.0×10^{9}	1.0×10^{3}	6.3	1.0×10^{3}	6.3	1.0×10^{3}	6.3	1.0×10^{3}	6.3	2.1×10^{9}	1.0×10^{3}	6.32
E. coli	0.5	$2.17\cdot10^{9}$	1.0×10^{3}	6.33	1.0×10^{3}	6.33	1.0×10^{3}	6.33	2.0×10^{5}	4.04	2.7×10^{9}	1.0×10^{3}	6.43	1.0×10^{3}	6.43	1.0×10^{3}	6.43	$4\cdot10^{3}$	5.83	2.1×10^{9}	1.0×10^{3}	6.32
ATCC 11229	3.0	$3.0\cdot10^{9}$	1.0×10^{3}	6.48	1.0×10^{3}	6.48	1.0×10^{3}	6.48	1.0×10^{3}	6.48	2.6×10^{9}	1.0×10^{3}	6.41	1.0×10^{3}	6.41	1.0×10^{3}	6.41	1.0×10^{3}	6.41	2.0×10^{9}	1.0×10^{3}	6.3
	5.0	$3.0\cdot10^{9}$	1.0×10^{3}	6.48	1.0×10^{3}	6.48	1.0×10^{3}	6.48	1.0×10^{3}	6.48	2.5×10^{9}	1.0×10^{3}	6.4	1.0×10^{3}	6.4	1.0×10^{3}	6.4	1.0×10^{3}	6.4	1.95×10^{9}	1.0×10^{3}	6.29
P. aeruginosa	0.5	$1.17\cdot10^{9}$	1.0×10^{3}	6.07	1.0×10^{3}	6.07	1.0×10^{3}	6.07	$6.2\cdot10^{5}$	3.28	1.0×10^{9}	1.0×10^{3}	6.0	1.0×10^{3}	6.0	1.0×10^{3}	6.0	$4.0\cdot10^{6}$	2.4	2.5×10^{9}	1.0×10^{3}	6.4
ATCC 15412	3.0	$1.17\cdot10^{9}$	1.0×10^{3}	6.07	1.0×10^{3}	6.07	1.0×10^{3}	6.07	1.0×10^{3}	6.07	1.0×10^{9}	1.0×10^{3}	6.0	1.0×10^{3}	6.0	1.0×10^{3}	6.0	$1.0\cdot10^{5}$	4.0	2.4×10^{9}	1.0×10^{3}	6.38
	5.0	$1.17\cdot10^{9}$	1.0×10^{3}	6.07	1.0×10^{3}	6.07	1.0×10^{3}	6.07	1.0×10^{3}	6.07	1.0×10^{9}	1.0×10^{3}	6.0	1.0×10^{3}	6.0	1.0×10^{3}	6.0	1.0×10^{3}	6.0	2.5×10^{9}	1.0×10^{3}	6.4
C. albicans	0.5	$3.77\cdot10^{8}$	1.0×10^{3}	5.58	1.0×10^{3}	5.04	$5.0\cdot10^{4}$	3.88	$1.0\cdot10^{6}$	2.58	8.0×108	1.0×10^{3}	5.9	1.0×10^{3}	5.9	1.0×10^{3}	5.9	$1.0\cdot10^{6}$	2.9	2.8×10^{8}	$6.5\cdot10^{5}$	2.64
ATCC 10231	3.0	$3.1\cdot10^{8}$	1.0×10^{3}	5.49	1.0×10^{3}	5.49	1.0×10^{3}	5.49	1.0×10^{3}	5.49	8.0×108	1.0×10^{3}	5.9	1.0×10^{3}	5.9	1.0×10^{3}	5.9	$5.0\cdot10^{4}$	4.21	2.7×10^{8}	$1.5\cdot10^{4}$	4.25
	5.0	$3.1\cdot10^{8}$	1.0×10^{3}	5.49	1.0×10^{3}	5.49	1.0×10^{3}	5.49	1.0×10^{3}	5.49	8.0×108	1.0×10^{3}	5.9	1.0×10^{3}	5.9	1.0×10^{3}	5.9	1.0×10^{3}	5.9	2.65×10^{8}	1.0×10^{3}	5.42
