# Supplementary material for: Antimicrobial activity of polyhexamethylene guanidine phosphate in comparison to chlorhexidine using the quantitative suspension method
Source: Ann Clin Microbiol Antimicrob. 2015 Jul 17;14:36. doi: 10.1186/s12941-015-0097-x (PMC4504446; doi:10.1186/s12941-015-0097-x)
Supplement: Additional file 2: — Table S2. Anti-microbial activity of antiseptics against perio- and cariopathogens in the quantitative suspension method. [file 12941_2015_97_MOESM2_ESM.pdf]

Table S2. Anti-microbial activity of antiseptics against perio –and cariopathogens in the quantitative suspension method

Test-culture	Exposure time, (min)	Polyhexamethylene guanidine phosphate (PHMG-P) concentration, %									Chlorhexidine (CHX) concentration, %								
		Control	1.0		0.5		0.2		0.05		Control	1.0		0.5		0.2		0.05	
		CFU/ml	CFU/ml	RF	CFU/ml	RF	CFU/ml	RF	CFU/ml	RF	CFU/ml	CFU/ml	RF	CFU/ml	RF	CFU/ml	RF	CFU/ml	RF
A. actinomycetemcomitans (HK 1519)	0.5	1.15×10^{9}	1.0×10^{3}	6.06	$1.0\cdot10^{3}$	6.06	2.5×10^{4}	4.66	5.5×10^{5}	3.32	1.16×10^{9}	1.0×10^{3}	6.06	1.0×10^{3}	6.06	1.0×10^{3}	6.06	7.05×10^{4}	3.16
	3	1.15×10^{9}	1.0×10^{3}	6.06	$1.0\cdot10^{3}$	6.06	$1.0\cdot10^{3}$	6.06	1.0×10^{3}	6.06	1.16×10^{9}	1.0×10^{3}	6.06	1.0×10^{3}	6.06	1.0×10^{3}	6.06	1.0×10^{3}	6.06
	5	1.15×10^{9}	1.0×10^{3}	6.06	$1.0\cdot10^{3}$	6.06	$1.0\cdot10^{3}$	6.06	1.0×10^{3}	6.06	1.16×10^{9}	1.0×10^{3}	6.06	1.0×10^{3}	6.06	1.0×10^{3}	6.06	1.0×10^{3}	6.06
P. gingivalis (ATCC 33277)	0.5	9.72×10^{8}	1.0×10^{3}	5.99	1.0×10^{3}	5.99	1.0×10^{3}	5.99	4.0×10^{5}	3.39	7.21×10^{8}	1.0×10^{3}	5.86	1.0×10^{3}	5.86	1.0×10^{3}	5.86	2.5×10^{4}	4.46
	3	9.72×10^{8}	1.0×10^{3}	5.99	1.0×10^{3}	5.99	1.0×10^{3}	5.99	1.0×10^{3}	5.99	7.21×10^{8}	1.0×10^{3}	5.86	1.0×10^{3}	5.86	1.0×10^{3}	5.86	1.0×10^{3}	5.86
	5	9.72×10^{8}	1.0×10^{3}	5.99	1.0×10^{3}	5.99	1.0×10^{3}	5.99	1.0×10^{3}	5.99	7.21×10^{8}	1.0×10^{3}	5.86	1.0×10^{3}	5.86	1.0×10^{3}	5.86	1.0×10^{3}	5.86
L. acidophilus (NCTC 1723)	0.5	1.28×10^{7}	9.71×10^{6}	0.12	9.69×10^{6}	0.12	9.55×10^{6}	0.13	8.92×10^{6}	0.16	1.25×10^{7}	2.39×10^{6}	0.72	4.24×10^{6}	0.47	1.94×10^{6}	0.81	8.61×10^{6}	0.16
	3	1.28×10^{7}	6.95×10^{6}	0.27	7.97×10^{6}	0.21	8.64×10^{6}	0.17	7.38×10^{6}	0.24	1.25×10^{7}	5.0×10^{3}	3.40	3.17×10^{4}	2.60	2.0×10^{4}	2.80	3.85×10^{6}	0.51
	5	1.28×10^{7}	4.8×10^{6}	0.43	4.6×10^{6}	0.45	8.06×10^{6}	0.20	6.73×10^{6}	0.28	1.25×10^{7}	$1.0\cdot10^{3}$	4.10	$1.0\cdot10^{3}$	4.10	1.67×10^{3}	3.88	1.89×10^{6}	0.82
S. mutans (CCUG27624; IngBrit)	0.5	6.27×10^{8}	3.13×10^{6}	2.30	9.20×10^{6}	1.83	1.87×10^{7}	1.52	1.35×10^{8}	0.67	5.39×10^{8}	5.0×10^{4}	4.03	6.67×10^{4}	3.91	2.25×10^{5}	3.38	5.56×10^{7}	0.99
	3	6.27×10^{8}	1.0×10^{3}	5.80	1.0×10^{3}	5.80	1.0×10^{3}	5.80	1.75×10^{5}	3.55	5.39×10^{8}	1.0×10^{3}	5.73	1.0×10^{3}	5.73	1.0×10^{3}	5.73	1.0×10^{5}	3.73
	5	6.27×10^{8}	1.0×10^{3}	5.80	$1.0\cdot10^{3}$	5.80	1.0×10^{3}	5.80	1.0×10^{3}	5.80	5.39×10^{8}	1.0×10^{3}	5.73	1.0×10^{3}	5.73	1.0×10^{3}	5.73	1.0×10^{3}	5.73
